# Neural correlates of processing sentences and compound words in Chinese

**DOI:** 10.1371/journal.pone.0188526

**Published:** 2017-12-01

**Authors:** Talat Bulut, Yi-Hui Hung, Ovid Tzeng, Denise H. Wu

**Affiliations:** 1 Institute of Cognitive Neuroscience, National Central University, Zhongli, Taiwan; 2 Department of Speech and Language Therapy, Istanbul Medipol University, Istanbul, Turkey; 3 Haskins Laboratories, Yale University, New Haven, Connecticut, United States of America; 4 Institute of Neuroscience, National Yang Ming University, Taipei, Taiwan; 5 The Institute of Linguistics, Academia Sinica, Taipei, Taiwan; Hangzhou Normal University, CHINA

## Abstract

Sentence reading involves multiple linguistic operations including processing of lexical and compositional semantics, and determining structural and grammatical relationships among words. Previous studies on Indo-European languages have associated left anterior temporal lobe (aTL) and left interior frontal gyrus (IFG) with reading sentences compared to reading unstructured word lists. To examine whether these brain regions are also involved in reading a typologically distinct language with limited morphosyntax and lack of agreement between sentential arguments, an FMRI study was conducted to compare passive reading of Chinese sentences, unstructured word lists and disconnected character lists that are created by only changing the order of an identical set of characters. Similar to previous findings from other languages, stronger activation was found in mainly left-lateralized anterior temporal regions (including aTL) for reading sentences compared to unstructured word and character lists. On the other hand, stronger activation was identified in left posterior temporal sulcus for reading unstructured words compared to unstructured characters. Furthermore, reading unstructured word lists compared to sentences evoked stronger activation in left IFG and left inferior parietal lobule. Consistent with the literature on Indo-European languages, the present results suggest that left anterior temporal regions subserve sentence-level integration, while left IFG supports restoration of sentence structure. In addition, left posterior temporal sulcus is associated with morphological compounding. Taken together, reading Chinese sentences engages a common network as reading other languages, with particular reliance on integration of semantic constituents.

## Introduction

Humans’ ability to process sentences in an effortless manner is an extraordinary demonstration of the superior cognitive capacities distinguishing humans from other animal species. Because sentence comprehension requires the engagement and coordination of word recognition, syntactic parsing based on word category and morphosyntactic information, semantic integration, and pragmatic inferencing among other processes, how the brain achieves the complex task within a short time has attracted the interest of linguists, psychologists, and neuroscientists. Despite the extensive research on syntactic processing in Indo-European languages, the exact operations that enable sentence comprehension and their neural correlates are far from being fully understood. Given that there is much less research on Chinese sentence processing, its anatomical and functional mechanisms are largely unexplored.

To identify the neural correlates of sentence processing, one common strategy adopted by previous research is to contrast the neuronal signals associated with hearing or reading well-formed sentences to those associated with hearing or reading word lists in which the composing words do not form meaningful sentences [1]. For example, the functional magnetic resonance imaging (FMRI) technique was employed to compare the brain responses to sentences and to unstructured word lists [2]. The results revealed a neural network, including the frontal operculum within left inferior frontal gyrus (IFG) and bilateral temporal poles (i.e., the anterior part of temporal lobe, aTL), demonstrating higher activation to sentences than to word lists. Consistent with such findings, a magnetoencephalography (MEG) study found that both the left aTL and IFG, together with posterior superior temporal gyrus (pSTG) and ventral medial areas, showed stronger responses to a word embedded in a well-formed sentence 250 ms after the word onset than to the same word embedded in an unstructured word list [3]. The results were interpreted as implicating these regions in sentence-level combinatorics, although it is not clear which specific sentence-level operations are associated with these particular regions.

Despite the fact that various studies have all pointed to the involvement of left aTL, IFG, and/or pSTG in sentence processing [2–5], the operations supported by these neural substrates are still under debate. Some studies have argued that the function of left (sometimes bilateral) aTL (including the temporal pole and the anterior portion of the STG) is related to structural building of linguistic materials [6,7], whereas other studies have indicated that the responses in aTL are driven by compositional semantics [8] or by the combination/interaction of semantic and syntactic processing [9]. The function of left IFG is equally controversial. Some studies have found the activity of this region to be modulated by structural complexity of experimental stimuli [4,10], whereas other studies have found this regions to be sensitive to the demand of working memory [11]. As for left pSTG, although most of the studies have identified this region to be associated with the processing of structural complexity [4,5,11], in one theory it has been proposed that this region serves the function of retrieval of lexico-syntactic information [10].

Although contrasting well-formed sentences to unstructured word lists has been employed to investigate Dutch [10], English [2,6,7], French [12,13], and German [14], to our knowledge it has not been applied to examine the neural substrates underlying the processing of Chinese sentences. According to the theory of Universal Grammar [15,16], no major difference is expected to distinguish syntactic processing of the deep structure underlying Chinese and other languages. However, the lack of morphosyntactic cues and absence of morphological inflections in Chinese distinguish it from other Indo-European languages in a significant way. For instance, Chinese has a very limited number of grammatical markers (e.g., limited verb inflection such as tense, aspect, person, number), and there is no subject-verb agreement. In addition, for Chinese, lexical category and syntactic function are not transparent; that is, many verbs can also be used as nouns without morphological changes. For these reasons, it has been argued that syntax and semantics are closely inter-related and context-dependent in Chinese [17].

These properties of Chinese might result in certain differences in sentence processing between Chinese and Indo-European languages with relatively rich morphosyntax. However, the intriguing typological differences and the resulting neural mechanisms of sentence processing have not been investigated intensively with neuroimaging techniques. An exception is an FMRI study comparing reading in Chinese and English in a natural story reading task and a lexical decision task [18]. In addition to task-based differences, the authors revealed differences in reading networks in the two languages in the visual cortex, left middle temporal gyrus and ventral occipitotemporal regions bilaterally. As for sentence processing, the few previous neuroimaging studies investigating sentence-level processes in Chinese generally employed violation paradigms involving syntactic or semantic anomalies [19–22]. For instance, an FMRI experiment in which participants read Chinese phrases with syntactic or semantic violations reported activation in a largely overlapping network for semantic and syntactic violations including left mid-inferior frontal and mid-superior temporal cortices [17]. These findings were interpreted as evidence that syntactic processing is less independent from semantic processing in Chinese than in Indo-European languages.

Another event-related FMRI study used semantic and syntactic (word category) violations as stimuli to investigate the neural correlates of syntactic processing when participants read Chinese sentences and performed an acceptability judgment task [21]. It was found that left BA44 (IFG) showed greater activation for combined semantic and syntactic violations compared to that for semantic-only violations. Importantly, the activation in this area did not differ significantly between the congruous and semantic violation conditions, suggesting that this region did not respond to semantic processing and is involved in syntax only, as the authors inferred. Such inference is in line with a number of studies in other languages that implicated Broca’s area with syntactic violations in English [23], German [14], Hebrew [24,25] and Japanese [26].

The association of the activation of left IFG with syntactic processing in Chinese [21] has been contested by at least three later FMRI studies which examined the neural signals associated with semantic violations [19,20,22]. Because it was found that activation was induced in left IFG when semantic expectancy of sentence fragments was manipulated, these studies attributed the function of this region to semantic integration, rather than to syntactic processing. Although inconclusive yet, in general the dissociations observed between syntactic and semantic processes in Chinese corroborates other findings from Indo-European languages [23,27].

Previous literature including those adopting the syntactic violation paradigm [17,21] clearly shows that left IFG is critically involved in sentence processing. However, this region has also been associated with the processing of non-linguistic stimuli. For example, left IFG has been linked to structure building in music [28], processing structural hierarchy of mathematical stimuli [29], and merging number words in Chinese and French [30]. Nevertheless, other studies also reported selective or specialized response of left IFG to syntactic processing of linguistic stimuli [31,32]. Furthermore, it was even put forward that left IFG contributes to syntactic working memory rather than to the processing of syntactic structure per se [33].

In consideration of the contested roles of syntax and semantics in Chinese sentence processing, it is worthwhile to investigate how the neural correlates of these operations are involved in natural sentence reading. As described above, there were a limited number of neuroimaging studies that investigated sentence-level processes in Chinese, and these studies generally adopted the violation paradigm [19–22]. However, the violation paradigm with abnormal sentences may not reflect normal sentence processing mechanisms. Furthermore, the studies with the violation paradigm adopted semantic and syntactic acceptability judgments, which are metalinguistic in nature and may engage readers in different processing strategies. Therefore, the present study employed a passive reading task to engage participants in a reading process that is as natural as possible. The primary goal of the current study is to reveal the neural network associated with Chinese sentence processing in contrast to that associated with lexical-semantic processing of unstructured word lists and character lists. It is predicted that the contrast of sentences versus word and character lists would isolate semantic and syntactic combinatorial operations at the sentence level. These operations are predicted to engage especially left-hemispheric temporal regions as in previous literature in Indo-European languages [2,6,9]. The left IFG may not be activated by the contrast of sentences versus word and character lists, since it may be the case that this area “comes into play when the processing load increases” [1], for example when there is a syntactic or semantic violation [19–22] or structural complexity [34,35].

The contrast between word lists and character lists is predicted to reveal semantic and morphological combinatorial operations at the word level. Chinese words typically consist of two or more characters, although there are also single-character words. Historically, individual characters that conveyed word meaning were later compounded to form words consisting of more than one character. Many characters that make up compound words also have their own individual meanings in isolation. For instance, the Chinese word “飲料” (meaning *beverage*) consists of the characters “飲” (*yǐn*, meaning *drink* as a noun or *to drink* as a verb) and “料” (*liào*, meaning *material*). Therefore, it is hypothesized that the process of combining characters to form words is associated with areas of the brain involved in morphological processing of compound words. In this context, it may be argued that Chinese words comprising multiple characters are accessed and processed as a whole word, rather than compounding the individual characters. However, previous naming and lexical decision studies have generally shown that properties of individual characters making up compound words (e.g., frequency) affect processing of the compound words, suggesting that constituent characters are also accessed and analyzed when processing compound words in Chinese (see [36] for a review). This confirms that the contrast of word lists vs. character lists reflects the effect of compounding.

Previous neuroimaging studies on derivational morphology (used for word formation) and inflectional morphology (used for indicating grammatical function) have identified generally left-lateralized fronto-temporal areas [37]. For example, in a PET study comparing encoding of auditorily presented words in Finnish, the left IFG was found to show stronger activation for inflected compared to monomorphemic nouns [38]. Similarly, an FMRI study on Finnish inflectional morphology revealed stronger activation for inflected versus monomorphemic words in frontotemporal regions including left IFG and posterior superior temporal sulcus [39]. In another FMRI study, regularly inflected past tense forms (e.g., *stayed*) were associated with stronger activation compared to irregular past tense forms (e.g., *taught*) in frontotemporal regions including left IFG and bilateral superior temporal gyrus [40]. In an FMRI study on derivation and inflection in Italian, word derivation activated a largely left lateralized frontal (including IFG) and parietal regions, whereas verb inflection produced similar, but more circumscribed activation [41]. In German, too, processing of complex derivational morphology associated left IFG as well as bilateral temporo-occipital and right parietal areas [42].

Consistent with the previous findings of derivational and inflectional morphology from Indo-European languages, an FMRI study found similar activation for reading Chinese characters and words, both of which involved left frontal and temporal areas when participants covertly generated a word that was semantically related to the stimulus [43]. In addition, right parietal and occipital areas were also involved in such processing. Most of the previous studies in English and other European languages focused on derivational and inflectional morphology, but not on compounding as a morphological operation. Few studies conducted on compounding Chinese characters, on the other hand, employed tasks that are not part of normal reading process (such as word generation) [43]. For these reasons, the second goal of the present study is to investigate neural correlates of compounding in Chinese by comparing unstructured word lists and character lists in a passive reading task.

## Methods

This study was carried out in accordance with the Social and Behavioral Research Ethical Principles and Regulations of Research Ethics Committee of National Taiwan University with written informed consent from all subjects. All subjects gave written informed consent in accordance with the Declaration of Helsinki. The protocol was approved by the Research Ethics Committee of National Taiwan University.

### Participants

Twenty-four healthy, right-handed native Chinese speakers (11 females; aged from 19yrs to 29yrs, M = 23) took part in this experiment. All participants had normal or corrected-to-normal vision and had no history of neurological or psychiatric disorders. We also made sure that participants met further qualifications of participating in MRI experiments. Each participant received 750 TWD as compensation.

### Stimuli and procedure

As exemplified in Table 1 below, 60 Chinese sentences of various syntactic structures containing eight to sixteen characters were prepared. For every sentence, a word list which did not form any syntactic hierarchical structure was created by changing the order of the words. Similarly, for every sentence a character list which did not form any meaningful word through word-compounding (i.e., combining adjacent characters) was created by changing the order of the characters.

**Table 1:** Experimental conditions and example stimuli.

Conditions	Example Stimuli
**Sentence (N = 60)**	含糖飲料對健康是一個危害 contain sugar beverage to health CV a C hazard (Beverages containing sugar are a hazard to health)
**Unstructured Word List (N = 60)**	糖飲料健康一危害對個含是 sugar beverages health a hazard to C contain CV
**Unstructured Character List (N = 60)**	料糖健一飲康害對是個危含 material sugar healthy a drink peaceful harm to CV C danger contain

[C: Classifier; CV: Copular verb]

**Fig 1:**
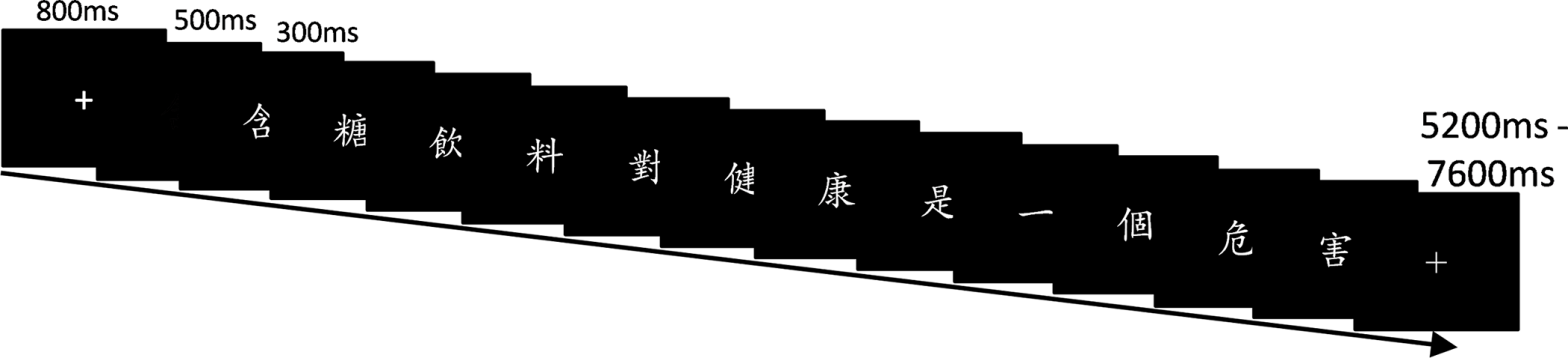
FMRI procedure.

An event-related experimental design was adopted as shown in Fig 1 below. Each trial started with a fixation cross followed by a blank screen. One character per frame was presented at the center of the screen for 300 ms without any inter-stimulus interval. Each Chinese character constituting the visual stimuli subtended a visual angle of approximately 1° vertically and was presented in DFKai-SB font in white against a black background. The trial ended with a final fixation, the length of which was varied between 5200 ms and 7600 ms, depending on the length of the trial. This variation was adopted in order to ensure that each trial lasted the same amount of time. The stimuli were presented with E-Prime software [44].

The experiment contained 6 runs, each one of which comprised 30 trials (10 trials randomly from each condition). The materials were Latin-squared and assigned to sessions such that the same characters were not shown more than once in two consecutive sessions. Six versions of materials were prepared in order to counter-balance the items and control for order of presentation. A passive reading task was adopted, in which 4 probes were randomly inserted in every run in order to ensure that participants paid attention to the stimuli. The probes consisted of a sentence asking participants to press either the left or the right button. In each run, there were equal number of probes requiring participants to press the left button and the right button. Each run took 7 minutes to complete. With a short break between runs, the entire experiment took about 45 minutes to finish.

### FMRI data acquisition

The MRI image was acquired with a Siemens MAGNETOM Skyra® 3T scanner (located in National Cheng-Chi University, Taipei, Taiwan) and a Siemens 32-channel whole-head coil. Participants’ heads were immobilized with a vacuum-beam pad in the scanner. A T2*-weighted gradient-echo echo planar imaging (EPI) sequence was used for FMRI scans, with slice thickness of 3.4 mm, no inter-slice gap, in-plane resolution of 3.4375 × 3.4375 mm, and TR/TE/flip angle = 2000 ms/40 ms/77^o^. The field-of-view was 220 × 220 mm, and the acquisition matrix is 64 × 64. Thirty-three oblique-axial slices were acquired to cover the whole brain. In all FMRI experiments, the first five volumes of each scanning run were discarded for signal equilibrium.

To obtain fine localization of the activities in FMRI experimental sessions, a high-resolution anatomical image of each participant’s whole brain using a T1-weighted, MPRAGE sequence (TR = 3500 ms/TE = 3.5 ms/flip angle = 90^o^) was obtained. The in-plane resolution of this image was 1 × 1 mm, and the slice thickness was 1 mm. The sequence took 192 slices along the axial planes of the brain, which took approximately 5 minutes to acquire.

### Data analysis

Functional MRI data were processed with the Statistical Parametric Mapping package (SPM8) developed by the Wellcome Trust Center for Neuroimaging at University of College London. Anatomical images were normalized to the standard brain template defined by the Montreal Neurological 152-brains average. In the preprocessing stage, the functional images were corrected for slice-timing differences, realigned to the first image of each run to correct for head movements, spatially normalized using the parameters obtained in the normalization of the anatomical images, resampled with a voxel size of 3×3×3 mm, and smoothed with a 5-mm Gaussian kernel. Experimental effects at each voxel were estimated using a multi-session design matrix. A general linear model (GLM) was created, including 4 trial types (3 conditions of the linguistic materials plus probe trials), each modeled by the canonical hemodynamic response function and its first-order time derivative, and 6 individual motion parameters to capture remaining signal variations due to head movements. The model also included high-pass filtering above 1/128 Hz. Individually estimated BOLD responses, smoothed with an 8-mm Gaussian kernel, were then entered into a one-way random-effect analysis with three conditions (sentence, word list, and character list) at the second level. An inclusive mask was created with ImCalc (formula: i1.*(i1>0)) on SPM8 software for each condition (sentence, word list, and character list). This mask served to mask out areas whose activation for the particular experimental condition did not exceed the activation for the baseline; hence, eliminating any activation between conditions that are due to negative values in comparison with the baseline. Application of this mask did not affect the *p*-values [45]. In addition to whole brain analysis, regions of interest (ROI) analysis was conducted on seven brain regions obtained from [30].

## Results

Four participants’ data were removed due to excessive misses on the probes (probe accuracy ≤75%). The remaining 20 participants’ probe accuracy was greater than 88% (mean accuracy = 98%, range: 88% - 100%).

### Whole brain analysis

**Fig 2:**
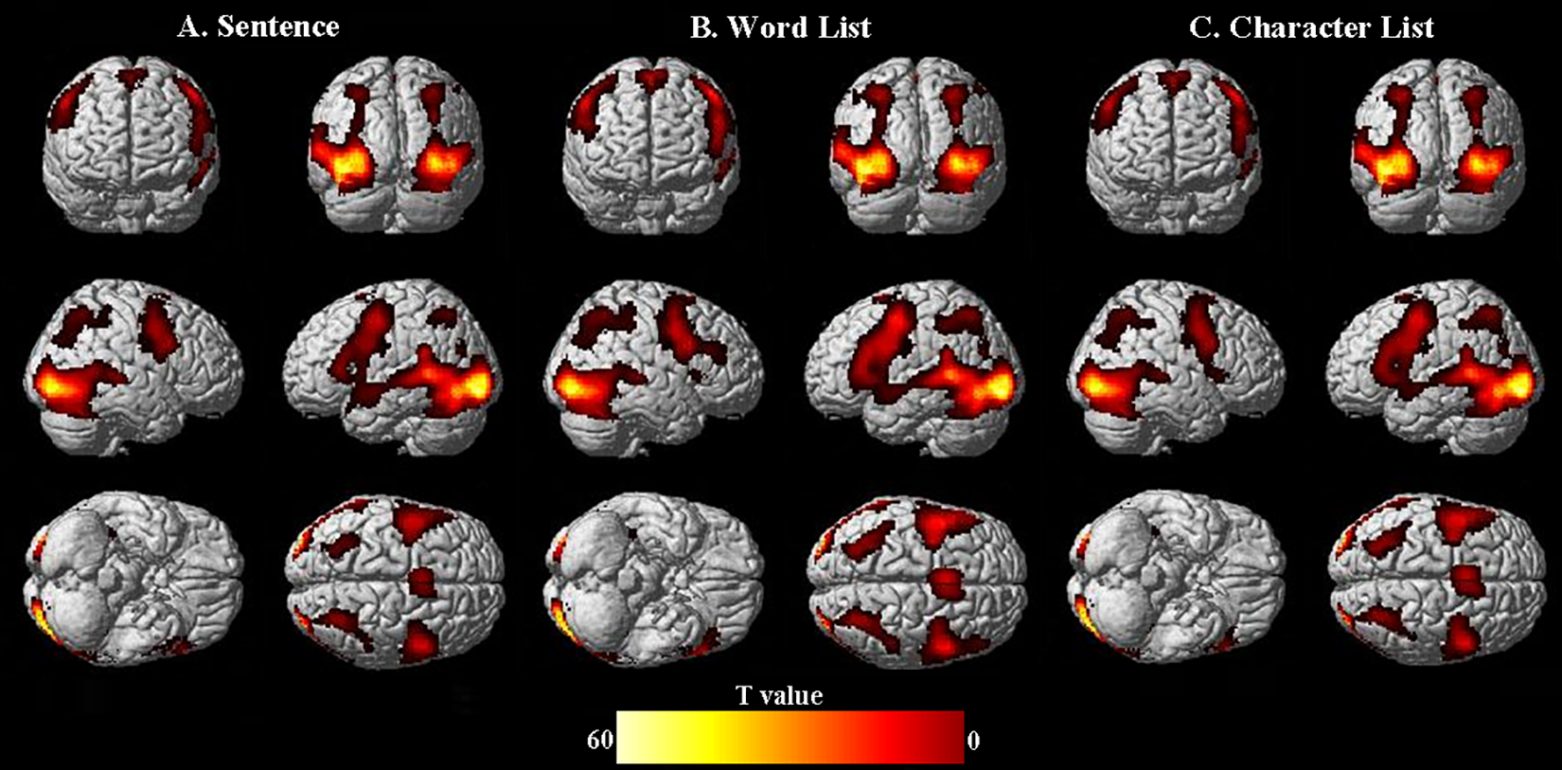
**Significant activations elicited by the main conditions of sentence (A), word list (B) and character list (C) against baseline (fixations & blanks).** Voxel-wise: uncorrected *ps* = 0.001; Cluster-wise: *ps* = FDR_*0*.*001*_.

**Fig 3:**
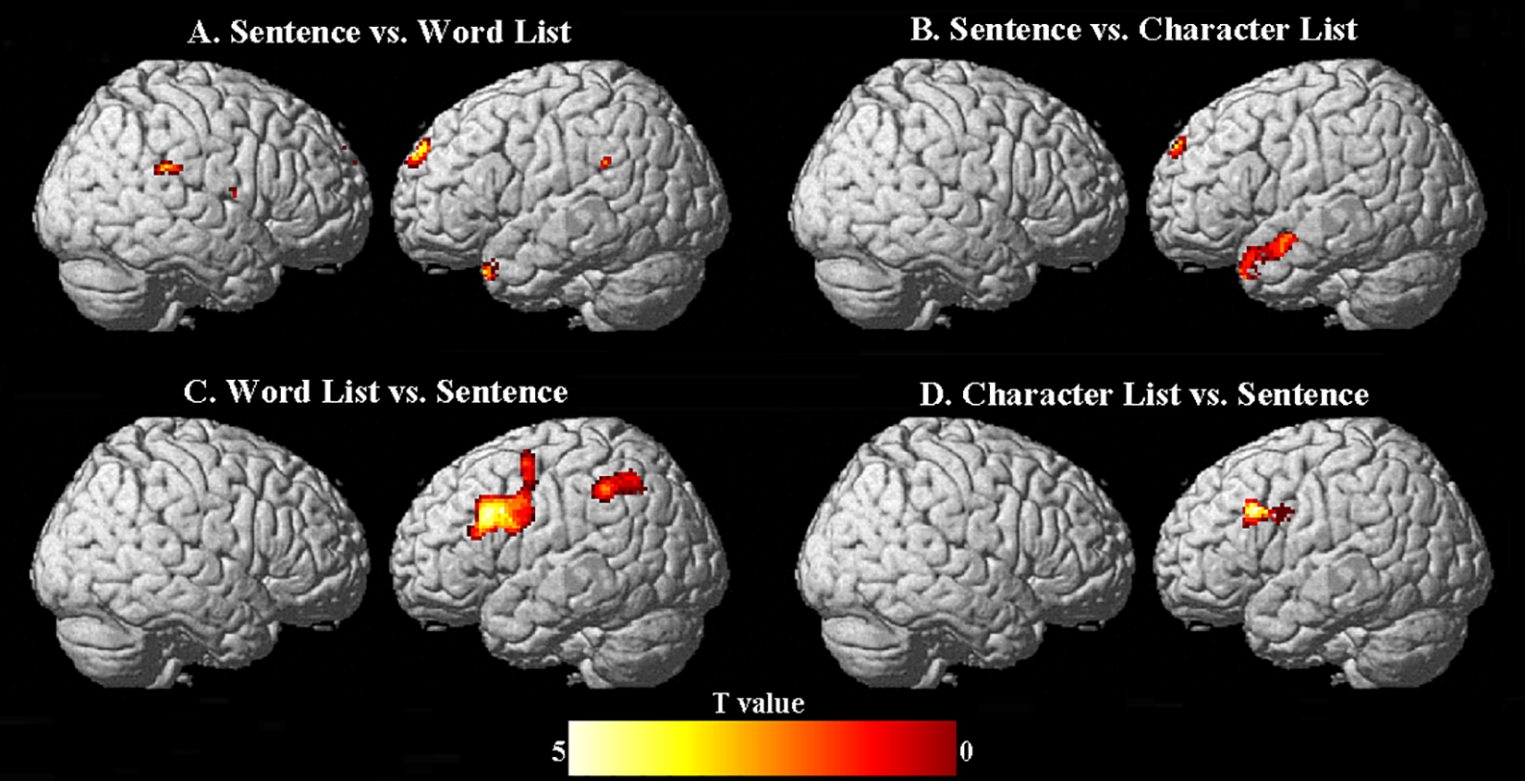
Significant activations elicited by each comparison. Panel A, B, D: uncorrected *ps* = .001; Panel C: Voxel-wise: uncorrected *p* = 0.001; Cluster-wise: *p* = FDR_*0*.*05*_.

As Fig 2 below shows, reading Chinese sentences and word/character lists activated bilateral perisylvian, inferior frontal and occipital regions, with stronger activation in the left hemisphere. This result is consistent with previous studies on Chinese reading [46]. Results of comparisons between the conditions are presented in Fig 3 and Table 2 below.

**Table 2:** Local activation maxima in MNI space for comparisons.

Comparisons	Anatomical Labels	Cluster Size	T Values	X	Y	Z
Sentence vs. Word List^a^	Pallidum_R	184	5.12	24	-8	-6
	Amygdala_L	108	4.67	-24	-8	-14
	Frontal_Sup_L	99	5.09	-16	56	36
	SupraMarginal_R	58	4.73	68	-32	26
	Temporal_Pole_Sup_L	39	4.26	-42	22	-32
	Rolandic_Oper_R	18	3.86	48	-2	12
	SupraMarginal_L	17	3.99	-60	-42	28
Sentence vs. Character List^a^	Temporal_Pole_Sup_L	204	4.00	-48	18	-24
	Frontal_Sup_L	45	4.92	-16	56	36
	Amygdala_L	34	4.11	-24	-6	-18
	Amygdala_R	12	3.64	22	-4	-18
Word List vs. Sentence^b^	Frontal_Inf_Tri_L	1372	5.44	-40	20	26
	Parietal_Inf_L	458	5.01	-44	-42	44
	Supp_Motor_Area_L	236	4.82	-6	16	54
	Insula_L	63	4.39	-28	24	6
	Insula_R	29	4.32	30	24	4
	Supp_Motor_Area_R	25	4.23	10	18	50
Character List vs. Sentence^a^	Frontal_Inf_Tri_L	288	4.18	-38	20	28
	Supp_Motor_Area_R	284	5.07	10	18	50
	Insula_R	46	3.86	30	24	4
	Insula_L	32	3.83	-32	24	6
(Sentence vs. Character List) ∩ (Word List vs. Character List)^c^	Temporal_Mid_L	12	2.42	-50	-34	0

^a^ Uncorrected *ps* = 0.001; k ≥ 10.

^b^ Voxel-wise: uncorrected *p* = 0.001; Cluster-wise: *p* = FDR_*0*.*05*_; k ≥ 10.

^c^ Conjunction analysis with uncorrected *p* = 0.001; k ≥ 10.

#### Sentence versus unstructured word list

This contrast, which putatively involves processing sentential semantics and syntax, revealed significant activation in a typical left lateralized frontotemporal network including left superior frontal gyrus and left superior temporal pole. Stronger activation was observed also in bilateral parietal lobes, specifically in the supramarginal gyrus. In addition to these cortical areas, sentence reading compared to word reading also involved subcortical regions; namely, right globus pallidus, left amygdala, and right rolandic operculum.

#### Sentence versus unstructured character list

This contrast, which entails both sentence-level semantic and syntactic processing as well as word-level morphological processing, showed significant activation in areas largely overlapping with the sentence versus word list contrast. In particular, a similar left-lateralized frontotemporal network was involved, including left superior temporal pole and left superior frontal gyrus as well as bilateral amygdalae. The contribution of left superior temporal pole increased markedly when the sentence condition was contrasted with the character list than with the word list condition. This may be attributed to greater combinatoric operations (at the word and sentence level) involved in the contrast between sentence versus character list than the contrast between sentence versus word list (only at the sentence level).

#### Unstructured word list versus unstructured character list

This contrast, which would isolate areas involved in morphological processing of Chinese words, did not reveal any significant activation at the adopted statistical threshold (uncorrected α = 0.001).

#### (Sentence vs. Character list) ∩ (Word list vs. Character list)

This conjunction analysis reflected the overlap between the two comparisons, which is morphological processing. In other words, the contrast of sentence vs. character list involves both sentence-level semantic and syntactic processing as well as word-level morphological processing, and the contrast of word list vs. character list entails morphological processing only. Thus, the intersection of these two contrasts would isolate morphological processing. This conjunction analysis revealed significant activation in left posterior middle temporal gyrus.

#### Unstructured word list versus sentence

This contrast yielded significant activation in a strongly left-lateralized frontoparietal network including the pars triangularis portion of left inferior frontal gyrus, bilateral supplementary motor area with greater cluster size in the left hemisphere, left inferior parietal lobule as well as bilateral insulae. It is argued that the word list versus sentence contrast might have caused participants to mentally rearrange unstructured word lists to arrive at the underlying message, thereby associating mostly left IFG (see Discussion).

#### Unstructured character list versus sentence

This contrast also revealed a mostly left lateralized frontal network with significant activation in the pars triangularis portion of left inferior frontal gyrus, right supplementary motor area, and bilateral insulae, which partially resembles the network involved with the word list versus sentence contrast. It may be the case that participants might have also rearranged unstructured character lists to reach the original sentential message, but to a lesser extent compared to unstructured word lists.

#### Unstructured character list versus unstructured word list

This contrast did not reveal any significant activation at the adopted statistical threshold (uncorrected α = 0.001).

In summary, the whole brain analyses implicated largely left-lateralized temporal and frontal regions with sentence level processing (sentence minus word/character list), while there was a word-level morphological/compounding effect in left posterior middle temporal gyrus, as revealed by the conjunction of sentence vs. character list and word list vs. character list. On the other hand, reverse contrasts (word/character list minus sentence) were associated mostly with the left inferior frontal gyrus as well as the left inferior parietal lobule.

### Region of interest analysis

For ROI analysis, seven regions of interest (IFG orbitalis, IFG triangularis, IFG opercularis, temporal pole (TP), anterior superior temporal sulcus (STS), posterior STS, temporo-parietal junction (TPJ)) were selected following one previous study on merging constituents in meaningful and meaningless French stimuli [4]. These regions were associated with merging linguistic constituents in French [4], and were also adopted by previous research in our lab to show that a subset of these regions are involved in merging numeric constituents in French and Chinese [30]. Since the present experiment also manipulated the size of linguistic constituents, with characters as individual constituents which can be combined into larger constituents as words and sentences, the same ROIs were used. Beta values from these seven ROIs plus their right homologs were subjected to a series of one-way ANOVAs with three levels (S-W-C) (Bonferroni corrected).

**Fig 4:**
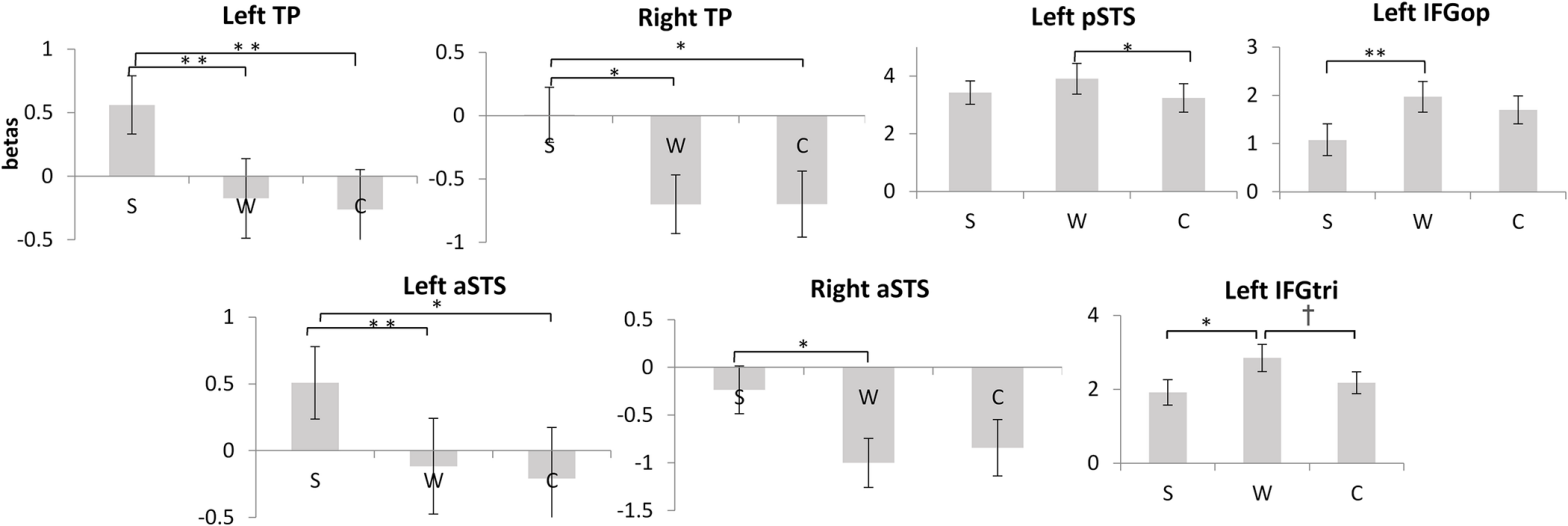
Significant beta values obtained in comparisons of Sentence (S), Words (W) and Characters (C). * *p* < .05; ** *p* < .01; † = .053 (Bonferroni corrected)]. [TP: Temporal Pole, IFGop: opercularis portion of inferior frontal gyrus, pSTS: posterior superior temporal sulcus, aSTS: anterior superior temporal sulcus, IFGtri: triangularis portion of inferior frontal gyrus.

As shown in Fig 4, significantly more activation was found for sentences compared to words and/or characters in temporal poles and anterior superior temporal sulci bilaterally, with a larger effect in the left hemisphere. Of these activations, those obtained in the right hemisphere were associated with negative beta values, showing that the activations were not larger than the baseline. However, the activations in the left hemisphere were associated with larger activations for sentences compared to the baseline. This anterior temporal network involved in sentence processing is consistent with the results of the whole brain analysis. Furthermore, more activation was found in the left posterior STS and left IFG triangularis for reading words compared to characters, which entails morphological processing of words. Finally, reading words led to stronger activation than reading sentences in left IFG opercularis and in left IFG triangularis, which arguably involves rearrangement of unstructured word lists to obtain the canonical sentence structure. There were no significant differences in the other ROIs in terms of beta values elicited by the experimental conditions, *ps* > .069 (Bonferroni corrected).

In summary, ROI analysis associated a largely left-lateralized network with the experimental conditions in line with the whole brain analysis. The temporal pole and anterior temporal regions were involved in sentence processing (sentence vs. word list), while part of left IFG and left posterior temporal region were associated with merging characters to form compound words (word list vs. character list). Lastly, left IFG again responded more strongly to word lists compared to sentences.

## Discussion

**Fig 5:**
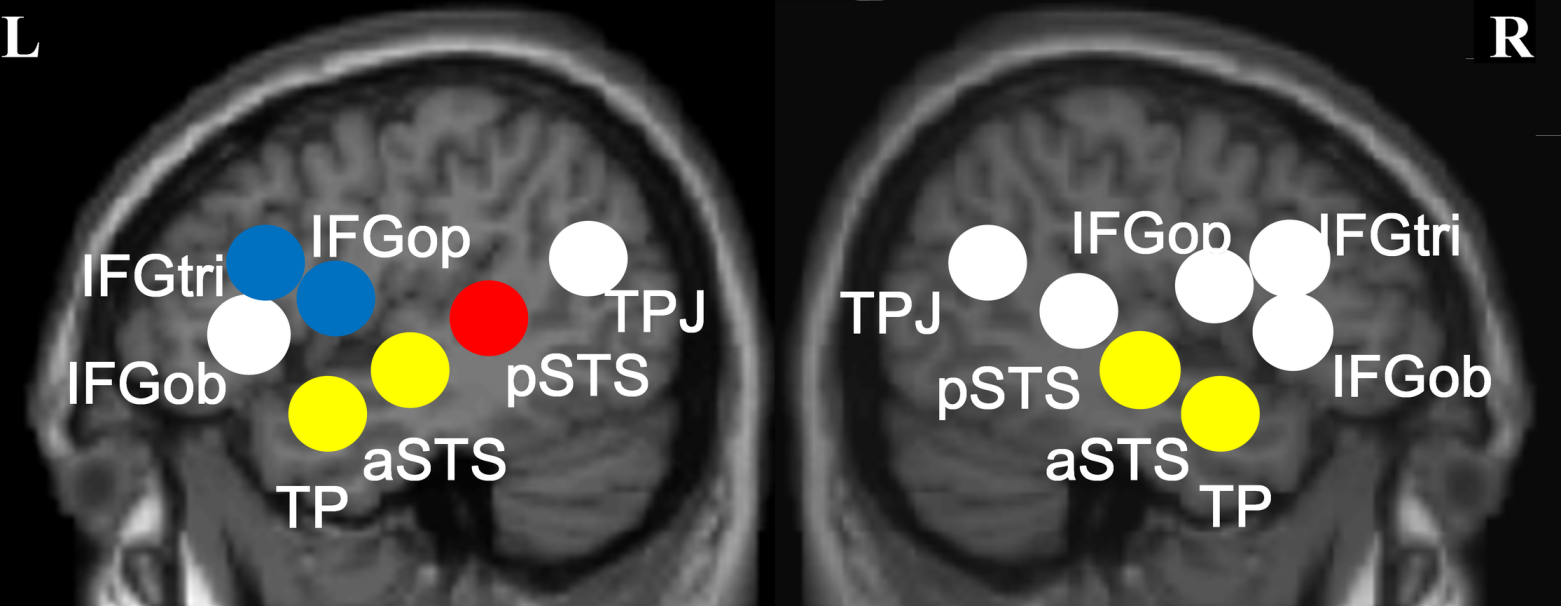
Regions of interest with their proposed functions. Yellow: Sentence-level integration (Sentence vs. Word/Character List); Red: Word-level integration/Compounding (Word List vs. Character List); Blue: Restoration of sentence structure (Word List vs. Sentence).

As summarized in Fig 5 below, the results associated reading Chinese sentences and word/character lists mainly with left-lateralized perisylvian areas. Specifically, sentences elicited more activation compared to both word and character lists in bilateral TPs and in left aSTS, whereas right aSTS showed more activation for sentences compared to words only. This finding is partially consistent with [2] which implicated bilateral temporal poles and left IFG in reading sentences compared to word lists. A MEG study also reported similar findings [3]; namely, both the left aTL and IFG, together with posterior superior temporal gyrus (pSTG) and ventral medial areas, showed stronger activation to a word embedded in a well-formed sentence than to the same word embedded in an unstructured word list. Based on the present findings, we conclude that especially left lateralized aTL is associated with integration of words in a sentential context in Chinese, which lacks morphosyntax and inflectional morphology, as previously suggested for other typologically different languages [4,8,11].

Our results that associated left anterior temporal regions with sentence processing (merging words to form sentences) overlap with the findings of Pallier and colleagues [4]. They found that increasing constituent size parametrically modulated activity in left IFG, anterior and posterior temporal regions and temporo-parietal junction. Importantly, this constituent size effect was limited to left IFG, including triangularis and opercularis portions, and posterior STS when the stimuli were delexicalized jabberwocky, hence associating these regions with purely structural constituent formation. On the other hand, when normal prose consisting of real French words were separately analyzed, constituent size effect was restricted to left TP, anterior STS and TPJ, showing that these regions respond to constituent size only in the presence of semantic information. Thus, in line with [4], it can be asserted that left TP and anterior STS may be involved in formation of semantic constituents in Chinese, as well. Intriguingly, left IFG appears not to be engaged in sentence processing for Chinese, which is not consistent with previous sentence processing studies in English [2,3]. Given that left inferior frontal regions have usually been associated with syntactic processing [34,35,47,48], absence of activation in this region for Chinese sentence reading per se suggests that Chinese reading largely depends on semantic processing [17]. However, when natural sentence reading process is disrupted, as in the case of syntactic violations, syntactic processing becomes necessary, hence the involvement of left IFG [21].

Although processing of Chinese sentences did not elicit stronger activation than processing of Chinese words/characters in left IFG, some parts of this region (i.e., pars triangularis and pars opercularis), as well as left IPL, responded more to word lists than to sentences, which seems rather surprising. As a possible explanation of this finding, the participants might have observed a syntactic violation when reading the unstructured word lists. This interpretation is in line with a number of studies in English [23] and Chinese [21] that associated left IFG with syntactic violations. Alternatively, this finding might also be related to restoration of the canonical sentence structure when reading the word lists. In the present experiment, word lists were created by scrambling the words in each sentence so that they do not form a structural unit. Because the words in word lists could be rearranged to restore the correct sequence, participants may have inadvertently reshuffled the words in their mind while reading word lists. To illustrate, when reading the isolated word list “sugar beverages health a hazard to contain are”, these words relate to the same semantic message; hence, in spite of their structural disarray, participants may have reordered the words mentally to obtain the original semantic message, which is “Beverages containing sugar are a hazard to health”.

Indeed, reordering of the words that can form natural sentences might have required more syntactic processing than reading natural sentences. This speculation is consistent with the findings from an FMRI study in German [49], which attributed argument hierarchy construction (determining the “actor” and “undergoer” of an action expressed by the verb) to left IFG (among other regions). Another FMRI study, in which the order of sentential arguments (subject and object) in German was varied, also associated pars opercularis of the left IFG with comprehending uncanonical object-first sentences compared with canonical subject-first sentences [50].

A number of neuroimaging studies in English have revealed involvement of left IFG in processing sentences with complex syntactic structures [34,35,47,48]. For instance, a position emission tomography (PET) study with a block design examined regional cerebral blood flow (rCBF) when participants read (center-embedded subject-modifying object relative clauses) and right-branching sentences (object-modifying subject relative clauses) while making acceptability judgments and non-word detection [35]. When judging from syntactic hierarchy, center-embedded sentences are with longer distance dependencies and more nested than right-branching ones. On the other hand, right-branching sentences are associated with more local syntactic operations than center-embedded ones. The results revealed extensive left hemispheric perisylvian activation for acceptability judgment, but not for non-word detection. Importantly, there was more activation for center-embedded structures than right-branching ones in the pars opercularis of Broca’s area. Consistently, a subsequent event-related FMRI study comparing different tasks associated the left IFG with syntactic processing of embedded object relative clauses compared to right-branching subject relative clauses [34]. Because left IFG revealed stronger activation for processing complex syntax even in a non-word detection task, the authors claimed task-independent involvement of this region in syntactic processing. Similarly, an FMRI study in Chinese also reported stronger activation in left IFG and posterior superior temporal gyrus for processing passive sentences compared to active sentences [51]. Because passive sentences are assumed by generative-transformational theories to be more complex than active sentences [52], the authors associated left IFG with syntactic movement processing in Chinese as in other Indo-European languages.

In addition to being linked to the processing of structurally complex sentences, left IFG has also been implicated to contribute to verbal short-term memory. For example, a PET experiment with a design comparable to the present study reported stronger activation in left IFG for reading word lists compared to reading simple sentences in Dutch [11]. Based on this finding, the authors claimed that left IFG may support a form of verbal working memory that maintains sentence structural information and lexical items [11,53]. Another study in German employed wh-questions to determine whether the activation in left IFG was modulated by syntactic integration costs (as contrasted between the structures of subject- and object-initial sentences) or by working memory load (as contrasted between the short- and long-distance of syntactic dependency) [33]. The FMRI results were more in line with the theory that left IFG contributes to syntactic working memory rather than to the processing of syntactic structure. In parallel with these studies in various Indo-European languages, it may be argued that for Chinese, too, left IFG is involved in processing syntactic complexity and/or syntactic working memory, which is required in reordering of the words when reading sentences with uncanonical word order.

It should be noted that the brain regions associated with sentence processing have also been demonstrated to be domain-general in some studies. In other words, the function of some of the sentence-related network (especially IFG) might be to build hierarchical structure in linguistic and other non-linguistic materials. For example, it was reported that left IFG (i.e., Broca’s area) responded more to musical sequences with incorrect chords than to sequences with correct chords, suggesting that this region is involved in structure building in music [28]. Similarly, it was observed that Broca’s area was commonly engaged in processing structural hierarchy of linguistic and mathematical stimuli [29]. Consistently, previous research in our lab reported a parametric effect of merging increasingly larger Chinese and French numeric constituents in left IFG and left IPL regions [30]. The present study also associated left IFG with compounding; that is, merging characters to form words, but not with merging words to form sentences, which elicited stronger activation in anterior temporal region. Furthermore, left IFG and left IPL revealed stronger activation for the contrast of word lists versus sentences, which was interpreted as restoration of the canonical sentence structure. Linguistically, these regions seem to be involved when normal reading process is disrupted in case of a violation or necessity to reorder the sentence fragments. Number processing, on the other hand, do not appear to entail temporal lobes since numbers are not as semantically rich as words, which is why only structural constituent formation takes place. Therefore, we argue that left IFG and left IPL are shared between linguistic and number processing domains in terms of purely structurally, but not semantically, driven constituent formation.

Despite such findings that suggest a non-specific role of left IFG in structural building of various domains, other studies found the same region to be selectively involved in syntactic processing but not in mathematical processing [31] or at least to be particularly efficient to process linguistic rather than other nonverbal structure [32]. Further evidence is definitely needed to determine whether the divergent results are due to individual differences of anatomical localization of this region across participants or due to different mechanisms being examined in different experimental paradigms across studies.

The whole brain analysis also revealed stronger activation in left IFG for character lists compared to sentences. This might reflect rearrangement of characters to form a semantically consistent message. However, this effect was not as strong as the effect observed for word list minus sentence contrast. Besides, left IPL, which was also activated for word lists compared to sentences, was not involved in the contrast of character list versus sentence. These results suggest that restoration of canonical sentence structure is not as salient in character lists as in word lists due to diminished structural and semantic cues that are locally available. For instance, the character list “material sugar healthy a drink peaceful harm to are danger contain” is not readily recombined to form the original semantic message. This difficulty to recombine the isolated characters is aggravated by the fact that in Chinese individual characters that make up words usually have their own lexical meanings in isolation. Hence, reduced activation in left IFG and no activation in left IPL associated with character list minus sentence contrast can be attributed to difficulty in recombining characters to form words and then sentences.

Another finding of the present study was that more activation was elicited in left pSTS for reading words compared to characters as revealed by the ROI analysis. In addition, the whole brain analysis also revealed an effect of morphological processing in left posterior temporal lobe, specifically in posterior middle temporal gyrus. The fact that reading words involved distinct cortical areas compared to reading characters contrasts with the findings of [43], which did not report any difference between reading Chinese words and single characters. This difference may stem from that study’s use of a word generation task for both stimulus types. Based on the present findings, we argue that left posterior temporal lobe, specifically left pSTS, is involved in compounding characters to form words/constituents. Similarly, Pallier and colleagues also associated left pSTS, but also left IFG, with merging linguistic constituents in French in the absence of semantic information and on the basis of purely structural information [4]. Accordingly, they claim that these regions can extract syntactic frames of a constituent on the basis of function words and morphological information alone. Along these lines, it may be argued that left pSTS supports the morphological process of merging characters to form compound words in Chinese. This finding is also partially consistent with neuroimaging studies on derivational and inflectional morphology in Finnish [38,39], English [40], Italian [41] and German [42], which, in general, involved posterior temporal regions as well as left IFG. It should also be noted that in addition to left pSTS, we also found more activation in left IFG triangularis, albeit with marginal significance, for reading disconnected words than characters. Given that in the present study both left IFG triangularis and opercularis were associated with structural operations, but only the former was involved, with marginal significance, in merging characters to form compound words, there may be a subdivision of left IFG in Chinese, which is certainly worth addressing in future studies.

From a linguistic point of view, the current findings are partially compatible with the neurobiological model of language processing called MUC (Memory, Unification, Control) proposed by Peter Hagoort [54,55]. The MUC framework does not place syntax in the center of the faculty of language as in generative approaches [15,16,56]. Instead, it is based on a tripartite architecture of the language system that includes phonological, syntactic and semantic/conceptual structures, in line with recent linguistic approaches advocated by Ray Jackendoff and colleagues [57–59]. According to MUC, a distinction is made between three functional components of language processing, namely, memory, unification and control, which are associated with distinct brain regions. The control component involves attentional and executive control processes required for appropriate social interaction, and recruits dorsolateral prefrontal cortex and the anterior cingulate cortex. The memory component, on the other hand, refers to representation of the linguistic knowledge including phonological, semantic, morphological, and syntactic building blocks of language. The MUC model associates the left temporal lobe as well as the angular gyrus of the parietal lobe with the memory component. In the current study, bilateral anterior temporal regions were found to be involved in sentence-level integration (sentence vs. word list), which may reflect retrieval of semantic and syntactic frames from memory, consistent with the MUC model. Furthermore, according to the model, the left posterior temporal lobe subserves retrieval of lexical information from memory, which is consistent with the current finding that pSTS is involved in word-level integration (word list vs. character list & sentence vs. character list). Unification refers to the process of deriving complex meanings and structures by combining the building blocks of language and is subserved by left IFG. Consistently, we reported stronger activation in pars opercularis and pars triangularis sections of left IFG for restoration of sentence structure (word list vs. sentence), which could be argued as indexing difficulty in unifying disordered words into a coherent sentence. Moreover, there was also stronger activation in pars triangularis of left IFG for word-level integration (word list vs. character list), which potentially reflects structural and semantic unification of individual characters to form words. However, it should be pointed out that sentence-level integration, which involves unification of syntactic and semantic information, did not reveal any significant activation in left IFG, contrary to the predictions of the MUC model. Previous studies in Chinese that reported IFG activation at sentence level generally used semantic and syntactic violations [19–22]. Therefore, it could be suggested that in Chinese left IFG is tasked when the sentence-level unification process is disrupted.

## Conclusion

In an event-related FMRI study with a passive reading task, neural correlates of processing Chinese sentences, unstructured word lists and unstructured character lists were explored. The results showed that when reading Chinese, which is characterized by limited morphosyntax and absence of inflectional morphology, a generally left lateralized network is activated. In particular, the anterior temporal lobe was found to respond more to reading Chinese sentences compared to reading unstructured word and character lists, indicating that this region may be responsible for integration at the sentence level. In addition, reading unstructured word lists elicited stronger activation in left posterior superior temporal sulcus compared to reading unstructured character lists, suggesting that this region may be involved in forming compound words out of individual characters. Finally, left IFG triangularis and opercularis as well as left inferior parietal lobule were activated more when reading unstructured word lists compared to sentences, which was interpreted as an effect of reordering sentence fragments and restoring sentence structure. Overall, the present findings indicate that reading Chinese sentences in a natural context only involves anterior temporal regions without implicating left IFG, which is partly different from sentence reading networks revealed by studies in English [2,3]. This difference may be attributed to the prominent role of semantics and diminished role of syntax in reading Chinese.
